# Self-compassion and its association with occupational burnout among young and middle-aged nurses: a cross-sectional study in China

**DOI:** 10.3389/fpsyg.2026.1905982

**Published:** 2026-07-24

**Authors:** Yanyan Dong, Qionghui Sun, Jiameng Shi, Yaohua Yu, Jianjun Zhu, Lifang Dong

**Affiliations:** 1 Ningbo College of Health Sciences, Ningbo, Zhejiang, China; 2 Ningbo No. 2 Hospital, Ningbo, Zhejiang, China; 3 Ningbo Medical Center Lihuili Hospital, Ningbo, Zhejiang, China; 4 The Affiliated Kangning Hospital of Ningbo University, Ningbo, Zhejiang, China

**Keywords:** cross-sectional study, emotional exhaustion, mindfulness, nursing workforce, occupational burnout, psychological resources, self-compassion, young and middle-aged nurses

## Abstract

**Objective:**

To investigate the current status of self-compassion and occupational burnout and to examine their correlation among young and middle-aged nurses aged 18–45 years.

**Methods:**

Using a combined purposive and convenience sampling strategy, a total of 564 young and middle-aged nurses aged 18–45 years from Zhejiang Province were enrolled between August 2024 and December 2024. Three instruments were administered: the Self-Compassion Scale (SCS), the Maslach Burnout Inventory-General Survey (MBI-GS), and a general demographic questionnaire.

**Results:**

The overall mean self-compassion score of participants was 3.41 ± 0.50. The dimension scores, ranked from highest to lowest, were as follows: self-kindness (3.65 ± 0.78), mindfulness (3.56 ± 0.79), self-judgment (3.46 ± 0.81), common humanity (3.33 ± 0.76), isolation (3.32 ± 0.80), and over-identification (3.14 ± 0.80). All SCS dimensions are scored such that a higher score indicates a higher level of that trait; for the total score, higher scores reflect lower self-compassion. The nurses exhibited mild-to-moderate occupational burnout, with a total mean score of 2.57 ± 1.18. The subscale scores in descending order were emotional exhaustion (3.02 ± 1.59), depersonalization (2.34 ± 1.78), and reduced personal accomplishment (2.20 ± 1.52). The total self-compassion score was significantly negatively correlated with the total occupational burnout score (r = −0.523, *p* < 0.001). Multiple linear regression analysis showed that self-compassion was a significant correlate of burnout, explaining 27.4% of the variance in total burnout scores.

**Conclusion:**

Young and middle-aged nurses demonstrated a moderate level of self-compassion overall. Self-compassion was negatively associated with occupational burnout, such that lower self-compassion corresponded to more severe burnout. These findings suggest that nursing administrators should implement strategies to enhance nurses’ self-compassion competence as a potential approach to mitigating occupational burnout.

## Introduction

With the rapid advancement of China’s healthcare system, public expectations for the quality of medical and nursing services continue to rise, and nurses are bearing increasingly heavy workloads and work-related stress. Prolonged high-intensity work, complex nurse–patient relationships, and multiple role conflicts render nurses susceptible to occupational burnout, which has emerged as a critical public health issue affecting their physical and mental well-being and the quality of nursing care (Pan et al., 2026; Zeng et al., 2020). According to a large-scale national survey, the prevalence of occupational burnout among clinical nurses in China ranges from 62.8 to 69.6%, a rate significantly higher than that of the general working population, underscoring the urgent need to address nurses’ occupational health (Wang et al., 2025; Yu et al., 2025).

Young and middle-aged nurses aged 18–45 years constitute the core workforce of clinical nursing teams, accounting for more than 70% of all registered nurses in China, and serve as the backbone driving the advancement of the nursing profession (Qin et al., 2023). This cohort is in a critical period of career establishment and progression, while simultaneously facing significant family responsibilities, making them particularly vulnerable to role conflicts and burnout. Therefore, focusing on this specific age group holds practical significance for workforce stability. Their occupational burnout not only affects personal physical and mental health and family harmony but also directly impacts the quality and efficiency of healthcare delivery. Burnout can lead to emotional exhaustion, diminished work engagement, and a detached, indifferent attitude toward patients and the work environment (Li et al., 2025), thereby impairing work performance and potentially causing medical errors and compromising patient safety (Hall et al., 2016). Studies (Wu F. et al., 2026; Alhazmi et al., 2026) have shown that higher levels of burnout are associated with stronger turnover intentions among nurses, which jeopardizes workforce stability and further exacerbates the shortage of nursing human resources.

Previous research (Westermann et al., 2014) has explored interventions for nurse burnout from the perspectives of the external work environment and multifaceted training, yet there is a lack of systematic correlational studies specifically focusing on the core group of young and middle-aged nurses, who may experience unique pressures, from the standpoint of internal psychological regulation. Self-compassion, a core construct in positive psychology, was introduced by Neff (2023b). It encompasses three pairs of opposing dimensions: self-kindness versus self-judgment, common humanity versus isolation, and mindfulness versus over-identification (Sengul et al., 2025). Self-compassion emphasizes treating one’s own inadequacies and suffering with a caring and understanding attitude, thus offering a novel direction for exploring internal correlates of burnout. According to the Conservation of Resources (COR) theory (Hobfoll, 1989), the psychological resources individuals possess are fundamental for coping with stress. As an internal psychological resource, self-compassion may help replenish emotional reserves, mitigate resource depletion, and consequently be associated with lower occupational burnout. Empirical evidence (Muris and Petrocchi, 2017) confirms that self-compassion is positively correlated with individual mental health, helping individuals enhance resilience, reduce emotional exhaustion, promote work–life balance, strengthen self-efficacy, and improve coping strategies (Şahin Tokatlıoğlu and Güner, 2026). While the link between self-compassion and burnout has been examined in healthcare professionals globally (Duarte et al., 2016; Dev et al., 2018), research focusing on the unique developmental stage of young and middle-aged nurses within the Chinese healthcare system remains limited.

Therefore, the present study focuses specifically on young and middle-aged nurses aged 18–45 years. Through a cross-sectional survey of 564 participants from Zhejiang Province, we aimed to investigate the status of self-compassion and occupational burnout in this population and to examine their association. The findings are expected to provide a theoretical basis for nursing managers to formulate targeted interventions and hold important practical significance for safeguarding the physical and mental health of young and middle-aged nurses and promoting their professional development.

## Subjects and methods

### Subjects

To recruit the specific population of young and middle-aged nurses, a combined purposive and convenience sampling method was employed. Young and middle-aged nurses meeting the eligibility criteria in Zhejiang Province were recruited from August 2024 to December 2024.

Inclusion criteria were: ① being a registered nurse with at least 1 year of work experience; ② aged 18–45 years; and ③ providing informed consent and voluntary participation. Exclusion criteria included nurses who had resigned, visiting staff, and those on extended leave. According to Kendall’s sample size estimation method (Pan et al., 2026), the sample size should be 5 to 15 times the number of variables. This study encompassed 12 dimensions, and a multiplier of 15 times the number of dimensions was adopted for preliminary sample size calculation. Anticipating an 80% valid questionnaire response rate, the required sample size was calculated to be 225 cases.

## Research instruments

### General information questionnaire

This questionnaire was designed by the research team and collected data on gender, age, marital status, educational level, professional title, hospital grade, clinical department, and whether the participant held an administrative position.

### Self-Compassion Scale (SCS)

The Self-Compassion Scale was originally developed by Neff (2023b) and subsequently translated and validated in Chinese by Chen et al. (2011) to measure self-compassion ability. The scale comprises six dimensions: self-kindness (5 items), self-judgment (5 items), common humanity (4 items), isolation (4 items), mindfulness (4 items), and over-identification (4 items), totaling 26 items. Self-kindness refers to being kind and understanding toward oneself in instances of suffering rather than being cold or judgmental; self-judgment refers to being harshly critical and disparaging toward oneself; common humanity refers to recognizing that imperfection and suffering are part of the shared human experience; isolation refers to feeling uniquely unfortunate, lonely, and different from others; mindfulness refers to holding painful emotions in balanced awareness without suppressing or exaggerating them; over-identification refers to becoming immersed in negative emotions and ruminating excessively (Neff, 2003a). Each item is rated on a 5-point Likert scale ranging from “never” to “always.” The scoring procedure is as follows: The negatively worded items of self-judgment, isolation, and over-identification are reverse-scored. Subsequently, a mean score for each dimension is calculated. The total SCS mean score is obtained by averaging the six dimension means. Consequently, a higher score on any dimension (including originally negative ones) indicates a higher level of that trait, and a higher total mean score indicates a lower overall level of self-compassion. The Chinese version of the SCS demonstrated a Cronbach’s alpha coefficient of 0.84 (He et al., 2023); in the present study, Cronbach’s alpha was 0.89.

### Occupational burnout scale

The Chinese version of the Maslach Burnout Inventory-General Survey (MBI-GS) (Chen et al., 2011) was employed. It includes three dimensions: emotional exhaustion (EE, 5 items), depersonalization (DP, 4 items), and reduced personal accomplishment (RPA, 6 items), totaling 15 items. Emotional exhaustion refers to feelings of being emotionally overextended and depleted of one’s work-related enthusiasm; depersonalization refers to maintaining a deliberate distance from the recipients of one’s work, adopting an indifferent and neglectful attitude toward the work context; reduced personal accomplishment refers to negative self-evaluation and a diminished sense of the meaning and value of one’s work (Lainidi et al., 2025). The questionnaire is scored on a 7-point Likert scale, with items rated from 0 (“never”) to 6 (“every day”). For EE and DP, a higher score indicates higher levels of exhaustion and cynicism, respectively. RPA items are reverse-scored, so a higher score on this dimension indicates a greater sense of reduced personal accomplishment. The total occupational burnout mean score is calculated as: 0.4 × EE + 0.3 × DP + 0.3 × RPA. A total score below 1.5 indicates no burnout; a score between 1.5 and less than 3.5 denotes mild-to-moderate burnout; and a score of 3.5 or above indicates severe burnout. Higher total scores reflect greater burnout severity (Luo et al., 2024). The Cronbach’s alpha of the MBI-GS in this study was 0.87.

### Investigation method

Data were collected via self-administered questionnaires. After receiving standardized training, the research team obtained approval from the nursing departments of the participating hospitals. Participants were selected strictly according to the inclusion and exclusion criteria. Upon obtaining informed consent, the survey was conducted by distributing electronic questionnaires online, and responses were collected anonymously. Standardized instructions were provided at the beginning of the questionnaire, requiring respondents to answer independently based on their actual circumstances; each IP address was restricted to one submission only. Questionnaires with clearly patterned or extreme responses were excluded as invalid. A total of 580 questionnaires were returned; after excluding 16 invalid ones, 564 valid questionnaires were retained, yielding an effective response rate of 97.24%.

### Statistical methods

Data were entered and analyzed using SPSS 26.0. Continuous data following a normal distribution were described as mean ± standard deviation (M ± SD), while categorical data were described using frequencies and percentages. Group comparisons were performed using independent samples t-tests, analysis of variance (ANOVA) with Welch’s correction applied when variances were unequal. The association between self-compassion and burnout was first examined using Pearson correlation analysis. Subsequently, a multiple linear regression analysis was performed to examine the independent association of self-compassion with burnout, controlling for demographic variables. Total occupational burnout score and its dimensions were treated as dependent variables in separate models. Preliminary analyses were conducted to ensure no violation of the assumptions of normality, linearity, multicollinearity, and homoscedasticity. 95% confidence intervals (CIs) for correlation coefficients and regression coefficients were calculated. The significance level was set at *α* = 0.05.

## Results

### General characteristics of participants and comparison of self-compassion scores by characteristics

The general characteristics of the participants are detailed in Table 1. There was a statistically significant difference in self-compassion scores among young and middle-aged nurses across different hospital grades (*p* < 0.05).

**Table 1:** General characteristics of participants and comparison of self-compassion total scores among young and middle-aged nurses with different characteristics (*n* = 564).

Variable	*n*	Total self-compassion score (Mean ± SD)	Test statistic	*P*
Gender			t = −0.174	0.864
Male	17	3.38 ± 0.66		
Female	547	3.41 ± 0.49		
Age (years)			*F* = 1.987	0.138
≤ 25	75	3.43 ± 0.49		
26–35	246	3.36 ± 0.49		
36–45	243	3.45 ± 0.50		
Marital status			*F* = 1.704	0.183
Unmarried	183	3.35 ± 0.47		
Married	369	3.44 ± 0.49		
Divorced/separated	12	3.43 ± 0.91		
Educational level			*F* = 0.546	0.580
Junior college	77	3.36 ± 0.45		
Undergraduate	472	3.42 ± 0.50		
Master’s	15	3.40 ± 0.41		
Professional title			*F* = 1.909	0.149
Primary	243	3.37 ± 0.46		
Intermediate	280	3.43 ± 0.52		
Senior	41	3.50 ± 0.52		
Hospital grade			*F* = 3.137	0.044*
Tertiary Grade A	390	3.44 ± 0.51		
Tertiary Grade B	122	3.32 ± 0.46		
Other	52	3.36 ± 0.44		
Department			*F* = 0.616	0.541
Internal medicine	196	3.39 ± 0.48		
Surgery	177	3.40 ± 0.52		
Other	191	3.44 ± 0.49		
Administrative position			t = 1.151	0.250
Yes	48	3.49 ± 0.48		
No	516	3.40 ± 0.50		

*p < 0.05. The total self-compassion score (SCS) was calculated such that a higher score indicates lower overall self-compassion. For gender and administrative position, independent samples t-tests were used; t-values are reported. For all other categorical variables, one-way analysis of variance (ANOVA) was used; F-values are reported.

### Scores of self-compassion and occupational burnout

The overall mean total self-compassion score for the young and middle-aged nurses was 3.41 ± 0.50. The dimension mean scores, ranked from highest to lowest, were: self-kindness (3.65 ± 0.78), mindfulness (3.56 ± 0.79), self-judgment (3.46 ± 0.81), common humanity (3.33 ± 0.76), isolation (3.32 ± 0.80), and over-identification (3.14 ± 0.80). The mean total occupational burnout score was 2.57 ± 1.18. The dimension scores in descending order were: emotional exhaustion (3.02 ± 1.59), depersonalization (2.34 ± 1.78), and reduced personal accomplishment (2.20 ± 1.52). See Table 2.

**Table 2:** Descriptive statistics of self-compassion and occupational burnout among young and middle-aged nurses (*n* = 564).

Variable	Score (Mean ± SD)
Self-Compassion (SCS)	
Total self-compassion score	3.41 ± 0.50
Self-kindness	3.65 ± 0.78
Mindfulness	3.56 ± 0.79
Self-judgment	3.46 ± 0.81
Common humanity	3.33 ± 0.76
Isolation	3.32 ± 0.80
Over-identification	3.14 ± 0.80
Occupational Burnout (MBI-GS)	
Total occupational burnout score	2.57 ± 1.18
Emotional exhaustion (EE)	3.02 ± 1.59
Depersonalization (DP)	2.34 ± 1.78
Reduced personal accomplishment (RPA)	2.20 ± 1.52

For the SCS, all negatively worded items (self-judgment, isolation, over-identification) were reverse-scored. All dimension and total scores are computed so that a higher score indicates a higher level of the named construct (e.g., a higher self-judgment score indicates greater self-judgment, and a higher total SCS score indicates lower overall self-compassion). For the MBI-GS, RPA items were reverse-scored; thus, a higher RPA score reflects a greater sense of reduced personal accomplishment. The total burnout score was calculated as 0.4 × EE + 0.3 × DP + 0.3 × RPA.

### Correlation between self-compassion and occupational burnout

Pearson correlation analysis (Table 3) revealed that the total self-compassion score was significantly negatively correlated with the total occupational burnout score among young and middle-aged nurses (r = −0.523, *p* < 0.001). Regarding burnout dimensions, emotional exhaustion was negatively correlated with mindfulness, self-judgment, isolation, and over-identification. Depersonalization was negatively correlated with self-kindness, mindfulness, self-judgment, isolation, and over-identification. Reduced personal accomplishment was negatively correlated with self-kindness, mindfulness, and common humanity. Detailed data, including 95% confidence intervals, are presented in Table 3.

**Table 3:** Correlations between self-compassion dimensions and occupational burnout among young and middle-aged nurses (r, 95% CI, *n* = 564).

Variable	Total MBI-GS	EE	DP	RPA
Total SCS	−0.523 (−0.59, −0.46)**	−0.376**	−0.410**	−0.351**
Self-kindness	−0.247 (−0.33, −0.16)**	−0.046	−0.096*	−0.463**
Mindfulness	−0.305 (−0.39, −0.22)**	−0.095*	−0.148**	−0.483**
Self-judgment	−0.350 (−0.43, −0.27)**	−0.334**	−0.341**	−0.040
Common humanity	−0.189 (−0.27, −0.11)**	−0.027	−0.074	−0.366**
Isolation	−0.435 (−0.51, −0.36)**	−0.443**	−0.428**	−0.008
Over-identification	−0.435 (−0.51, −0.36)**	−0.453**	−0.442**	0.021

*P < 0.05; **P < 0.001. SCS, Self-Compassion Scale; MBI-GS, Maslach Burnout Inventory-General Survey; EE, Emotional Exhaustion; DP, Depersonalization; RPA, Reduced Personal Accomplishment. 95% confidence intervals (CIs) are presented alongside the correlation coefficients for the total MBI-GS score. Scoring directions for all scales are consistent with the notes in Table 2.

Multiple linear regression analysis (Table 4) showed that, after controlling for significant demographic variables (hospital grade), the total self-compassion score remained a significant negative correlate of total burnout (*β* = −0.512, *p* < 0.001), explaining an additional 26.1% of the variance (ΔR^2^). In the final model, self-compassion and hospital grade together accounted for 27.4% of the variance in total occupational burnout (Adjusted R^2^ = 0.274). Similar patterns were observed when examining burnout subscales as dependent variables, with self-compassion being a significant correlate for emotional exhaustion, depersonalization, and reduced personal accomplishment (*p* < 0.001).

**Table 4:** Multiple linear regression analysis of factors associated with total occupational burnout among young and middle-aged nurses (*n* = 564).

Variable	B	SE	β	t	*P*	95% CI for B
Step 1						
(Constant)	2.09	0.12		17.83	<0.001	(1.86, 2.32)
Hospital Grade (Ref: Tertiary Grade A)						
Tertiary Grade B	0.23	0.12	0.08	1.92	0.055	(−0.01, 0.47)
Other	0.35	0.15	0.10	2.42	0.016	(0.06, 0.64)
Step 2						
(Constant)	6.75	0.34		19.85	<0.001	(6.08, 7.42)
Hospital Grade (Ref: Tertiary Grade A)						
Tertiary Grade B	0.13	0.10	0.04	1.24	0.215	(−0.07, 0.33)
Other	0.24	0.12	0.07	1.94	0.052	(−0.00, 0.48)
Total SCS score	−1.21	0.09	−0.51	−13.44	<0.001	(−1.39, −1.03)

R^2^ = 0.015 for Step 1; ΔR^2^ = 0.261 for Step 2 (*P* < 0.001). Total model R^2^ = 0.276, Adjusted R^2^ = 0.274. Abbreviations: SCS, Self-Compassion Scale. The total SCS score is scored such that higher values indicate lower self-compassion; therefore, the negative coefficient indicates that lower self-compassion (higher SCS score) is associated with higher burnout. CI, confidence interval.

## Discussion

The results of this study indicate that the self-compassion of young and middle-aged nurses, as a whole, is at a moderate level (Yu and Gui, 2022). This finding aligns with conclusions from domestic research on critical care nurses (Liu et al., 2025), and is comparable to levels reported among nurses in other international settings (Duarte et al., 2016), suggesting that there remains considerable room for improvement in self-compassion among clinical nurses in China. Regarding the distribution of dimension scores, self-kindness scored the highest, whereas over-identification scored the lowest. The descending order of scores—self-kindness, mindfulness, self-judgment, common humanity, isolation, and over-identification—reflects that these nurses can still face work pressures and personal shortcomings with a degree of understanding. However, they exhibit deficits in emotional regulation, tend to become entangled in negative emotions, and lack effective mindful awareness. Analysis of demographic characteristics revealed a significant difference in self-compassion scores among nurses working in hospitals of different grades. Those in Tertiary Grade A hospitals had the highest self-compassion scores, which may be attributable to disparities in resource allocation across hospital tiers. Tertiary Grade A hospitals generally place greater emphasis on professional development and the maintenance of staff mental health, offering more psychological support training and career development resources. Consequently, their nurses have greater exposure to humanistic care and psychological adjustment training, facilitating relatively higher self-compassion. Conversely, primary hospitals, constrained by limited human and material resources, pay insufficient attention to nurses’ mental health and lack systematic psychological intervention measures, leading to comparatively lower self-compassion (Luo et al., 2024). This finding underscores the important influence of hospital resource allocation and organizational culture on the development of nurses’ self-compassion and implies that nursing managers should attend to the balanced advancement of mental health support across different levels of hospitals.

This study found that young and middle-aged nurses experienced mild-to-moderate occupational burnout, indicating that a certain degree of burnout is prevalent in this population and warrants considerable attention from nursing managers. In terms of dimension scores, emotional exhaustion scored the highest, followed by depersonalization and reduced personal accomplishment. This pattern is consistent with existing evidence that the most prominent manifestation of burnout among these nurses is the excessive depletion of emotional resources (Yu and Gui, 2022). As the core workforce, nurses aged 18–45 years engage in long-term, high-intensity clinical nursing work, continuously providing emotional support and professional care to patients. Their emotional resources are persistently expended and cannot be promptly replenished (Liu et al., 2025). At the same time, nurses in this age group face multiple concurrent stressors, including career advancement, professional title promotion, family obligations, and child-rearing. The resulting work–family conflict further intensifies emotional exhaustion, corroborating the highest score observed in the emotional exhaustion dimension (Wu C. B. et al., 2026). Depersonalization scored second highest, suggesting that under the influence of prolonged emotional exhaustion, some nurses begin to exhibit indifference and deliberately maintain a detached distance from care recipients as a maladaptive coping strategy (Zhang et al., 2026). This requires timely intervention to prevent the further progression of burnout. The score for reduced personal accomplishment was relatively lower, indicating that despite emotional exhaustion, most young and middle-aged nurses still maintain fundamental recognition of the value of the nursing profession. On the one hand, nurses in this age group are in a phase of career establishment and bear household financial responsibilities, and their original vocational commitment helps them identify with professional values (Tan et al., 2025). On the other hand, the healthcare sector’s notable job security amid a challenging macroeconomic climate further facilitates their endorsement of nursing’s professional worth.

The results demonstrated a significant negative correlation between self-compassion and occupational burnout (*p* < 0.001). The multiple regression analysis further confirmed that lower self-compassion scores were robustly associated with higher burnout, even after accounting for relevant demographic factors. This finding provides empirical support for the core tenet of Conservation of Resources theory (Zhou et al., 2026; Ying et al., 2026)—that an individual’s psychological resources are fundamental for coping with work stress. When individuals face persistent work demands, they continuously expend psychological resources; if resource consumption exceeds replenishment, occupational burnout ensues (Halbesleben, 2006). As a critical internal psychological resource, self-compassion may help individuals supplement emotional reserves and reduce resource drain in stressful situations, thereby potentially preventing and alleviating burnout (Raab, 2014; Aşıklı et al., 2026). The cross-sectional nature of our data, however, precludes a definitive causal interpretation. Young and middle-aged nurses who become trapped in negative emotions, experience helpless loneliness, and habitually engage in self-judgment are prone to burnout (Gurzyńska et al., 2026). In contrast, burnout symptoms are less severe among those who treat themselves kindly, mindfully perceive their emotions, and recognize that occupational setbacks represent common human experiences rather than personal failings (Profit et al., 2014). These patterns are consistent with findings from similar studies conducted in Western and other Asian healthcare settings, suggesting a potentially universal mechanism (Duarte et al., 2016; Neff, 2023b). These insights suggest that nursing managers can provide targeted education on self-acceptance and common humanity, as well as mindfulness and emotion regulation training for nurses exhibiting severe burnout. Establishing a supportive work environment that helps reduce rumination over negative feelings, isolation, and self-judgment is essential. Enhancing nurses’ self-compassion may serve as a strategy to replenish their psychological resources, be associated with lower burnout, contribute to stabilizing the nursing workforce, and ultimately improve clinical service quality.

## Limitations

Several limitations of the present study should be acknowledged when interpreting the findings. First, the study employed a cross-sectional design, which precludes any causal inference regarding the relationship between self-compassion and occupational burnout. Although the results demonstrate a significant negative correlation, the directionality of this association cannot be definitively established; it remains plausible that higher burnout erodes self-compassion capacities, or that a bidirectional relationship exists (Olson et al., 2015). Longitudinal or experimental designs are necessary to disentangle the temporal dynamics and causal pathways between these constructs. Second, the sample was recruited using a combined purposive and convenience sampling method from hospitals in a single province of China, which limits the geographical representativeness and generalizability of the findings to young and middle-aged nurses in other regions or healthcare systems with differing cultural, organizational, and resource contexts (Andrade, 2021; Jiang et al., 2023). Multi-center, nationally representative studies are warranted to validate and extend these results. Third, the sample was overwhelmingly female (97%), reflecting the gender demographics of the nursing profession in China but severely limiting the generalizability of the findings to male nurses. Future research should include a more balanced sample to explore potential gender differences in the self-compassion-burnout association (Jager et al., 2017). Fourth, all data were collected via self-report questionnaires, which are susceptible to common method bias and social desirability effects. Participants may have underreported burnout symptoms or overestimated their self-compassion due to concerns about professional image or self-presentation, potentially attenuating or inflating the observed associations (Edwards et al., 2026). Future research would benefit from incorporating objective or multi-source indicators of burnout, such as physiological markers, absenteeism records, or supervisor ratings. Fifth, the study did not account for a range of potential confounding variables that could influence the self-compassion–burnout relationship, including personality traits, coping styles, workplace social support, workload intensity, and organizational justice perceptions (Podsakoff et al., 2003). The omission of these variables may obscure the true nature and magnitude of the association. Sixth, although self-compassion was treated as a global construct in this analysis, the differential patterns of association between specific self-compassion dimensions and burnout subscales suggest that examining the six dimensions separately could yield more nuanced insights (Voit, 2019). The current total-score approach may mask dimension-specific effects that carry distinct intervention implications. Finally, the reliance on a single time point of measurement did not permit an assessment of whether the relationship between self-compassion and burnout fluctuates across different career stages or in response to acute occupational stressors. Despite these limitations, the study contributes meaningful evidence on the correlational relationship between self-compassion and nurse burnout and offers a foundation for future research employing more rigorous designs and representative sampling.

## Conclusion

The self-compassion of young and middle-aged nurses is at a moderate level and is negatively associated with occupational burnout. The results indicate that nursing managers should prioritize cultivating self-compassion in this population, supplementing their internal psychological resources to potentially reduce burnout. Specifically, the findings highlight the need for differentiated strategies across hospital tiers, as nurses in primary hospitals reported lower self-compassion. Ultimately, this approach can promote nurses’ physical and mental health, stabilize the nursing team, and enhance the quality of nursing services. Several limitations of this study should be acknowledged. The sample was recruited via combined purposive and convenience sampling and drawn exclusively from Zhejiang Province; thus, the representativeness and generalizability of the findings require further enhancement. Future research should conduct multicenter, large-sample surveys to further validate these conclusions. Building on these findings, researchers should develop targeted self-compassion intervention programs and verify, through longitudinal interventional studies, the potential effects of self-compassion training on mitigating occupational burnout among nurses.

## Data Availability

The raw data supporting the conclusions of this article will be made available by the authors, without undue reservation.
